# Advances in Allogeneic Cancer Cell Therapy and Future Perspectives on “Off-the-Shelf” T Cell Therapy Using iPSC Technology and Gene Editing

**DOI:** 10.3390/cells11020269

**Published:** 2022-01-13

**Authors:** Yoshiki Furukawa, Yasuharu Hamano, Shuichi Shirane, Shintaro Kinoshita, Yoko Azusawa, Jun Ando, Hiromitsu Nakauchi, Miki Ando

**Affiliations:** 1 Department of Hematology, Juntendo University School of Medicine, Tokyo 113-8421, Japan; y-furukawa@juntendo.ac.jp (Y.F.); y-hamano@juntendo.ac.jp (Y.H.); sshirane@juntendo.ac.jp (S.S.); s-kinoshita@juntendo.ac.jp (S.K.); j-ando@juntendo.ac.jp (J.A.); 2 Division of Cell Therapy & Blood Transfusion Medicine, Juntendo University School of Medicine, Tokyo 113-8421, Japan; y.azusawa.ce@juntendo.ac.jp; 3 Division of Stem Cell Therapy, Distinguished Professor Unit, The Institute of Medical Science, The University of Tokyo, Tokyo 108-8639, Japan; 4 Institute for Stem Cell Biology and Regenerative Medicine, Stanford University School of Medicine, Stanford, CA 94305-5461, USA

**Keywords:** allogeneic cell cancer immunotherapy, CART therapy, donor lymphocyte infusion, gene editing, graft versus host disease, graft versus leukemia, induced pluripotent stem cells, inducible caspase-9, “off-the-shelf” T cell therapy, rejuvenated CTL, T cell exhaustion, virus-specific cytotoxic T lymphocytes

## Abstract

The concept of allogeneic cell therapy was first presented over 60 years ago with hematopoietic stem cell transplantation. However, complications such as graft versus host disease (GVHD) and regimen-related toxicities remained as major obstacles. To maximize the effect of graft versus leukemia, while minimizing the effect of GVHD, donor lymphocyte infusion was utilized. This idea, which was used against viral infections, postulated that adoptive transfer of virus-specific cytotoxic T lymphocytes could reconstitute specific immunity and eliminate virus infected cells and led to the idea of banking third party cytotoxic T cells (CTLs). T cell exhaustion sometimes became a problem and difficulty arose in creating robust CTLs. However, the introduction of induced pluripotent stem cells (iPSCs) lessens such problems, and by using iPSC technology, unlimited numbers of allogeneic rejuvenated CTLs with robust and proliferative cytotoxic activity can be created. Despite this revolutionary concept, several concerns still exist, such as immunorejection by recipient cells and safety issues of gene editing. In this review, we describe approaches to a feasible “off-the-shelf” therapy that can be distributed rapidly worldwide. We also offer perspectives on the future of allogeneic cell cancer immunotherapy.

## 1. Introduction

More than 60 years ago, in 1957, Dr. E. Donnall Thomas initially reported an innovative approach towards leukemia using radiation and chemotherapy followed by intravenous infusion of bone marrow [1,2], the first report of allogeneic hematopoietic stem cell transplantation (allo-HSCT), in which a donor’s hematopoietic stem cells and his or her immunological repertoires are infused into a patient to establish donor-derived hematopoiesis and immunity [3]. Thomas et al. conducted many laboratory and clinical investigations [4,5,6,7,8,9] that paved the way for current allo-HSCT, for which, in 1990, along with Dr. Joseph E. Murray, Thomas was honored with the Nobel Prize in Physiology or Medicine. Allogeneic cell therapy has continued to improve and has endured as an effective treatment option for both malignant and non-malignant diseases, especially for hematologic malignancies such as leukemia, which was formerly thought to be an incurable disease. Although allo-HSCT is a revolutionary treatment strategy, significant complications remain; most importantly, graft-versus-host disease (GVHD) and conditioning related toxicities.

The first report of GVHD was in 1959, when Billingham and colleagues described an immune reaction after bone marrow infusion, including skin rash and diarrhea, which they called “Runt Disease” [10]. GVHD is a life-threatening complication that occurs when immunocompetent T cells in donated tissue recognize the recipient as foreign, resulting in activation of donor T cells. These gain cytotoxic capacity and then attack the recipient to eliminate “foreign” antigen-bearing cells [11]. Management of both acute and chronic GVHD severely challenged early transplantation research groups. In 1974, the first grading system for acute GVHD was created [12], increasing awareness of skin, gastrointestinal, and liver symptoms in transplanted patients. In early trials, GVHD occurred in almost half of patients who received methotrexate prophylaxis. Major progress occurred when methotrexate was combined with calcineurin inhibitors such as cyclosporine or tacrolimus, initially in canine models and subsequently in patients, with improved overall survival [13,14,15,16]. Effective treatment for acute GVHD with antithymocyte globulin (ATG) was also reported [17], and these synergistic drug combinations of methotrexate, ATG, cyclosporine, and tacrolimus are still used to prevent GVHD [18]. Unfortunately, among patients undergoing allo-HSCT, 30–50% have acute GVHD (grade I–IV), and 14% have severe acute GVHD (grade III–IV) [11]. Risk factors include the degree of human leukocyte antigen (HLA) mismatch, unrelated donor, a female donor for a male recipient, the use of peripheral blood stem cell grafts, and myeloablative conditioning [19,20,21,22].

Chronic GVHD also remains as a major cause of morbidity and non-relapse mortality after allo-HSCT [23,24,25,26]. Approximately 30–70% of allo-HSCT recipients surviving at least 100 days after transplant develop chronic GVHD [26]. The skin is the most commonly affected site, with manifestations that can be observed in up to 75% of chronic GVHD patients, but clinical features may affect multiple organs or body areas with varying presentation, depending on the patient [27,28]. Corticosteroids with or without calcineurin inhibitors compose first-line treatment, but the prognosis is poor if the patient is steroid-refractory [29].

Other than GVHD, severe immunodeficiency is ubiquitous after allogeneic transplantation. Cytomegalovirus, Epstein-Barr virus (EBV), polyomavirus, human herpesvirus 6, and community-acquired respiratory viruses substantially affect allo-HSCT related morbidity and mortality [3,30]. Although drug therapy to prevent infection is indispensable [13,31] and treatments for infection are advancing, fatal infections are still inevitable following allo-HSCT.

The difficulty of treating GVHD is to balance graft versus leukemia (GVL), where the key to remission lies in maximizing the effect of GVL while minimizing the effect of GVHD. GVL was first hypothesized in 1979 when Weiden et al. reported that the risk of leukemia relapse was 2.5 times lower in patients with GVHD than in those without GVHD [32].

Many strategies to reinforce GVL have been deployed, such as donor lymphocyte infusion (DLI) [33] and natural killer cells [34,35]. DLI has augmented GVL effectively, particularly in multiple myeloma [36,37] and chronic myeloid leukemia [38]. However, DLI may cause GVHD so severe as to be life-threatening [39]. DLI has also been successfully used after allo-HSCT against severe viral infections, such as those with EBV and adenovirus, where adoptive transfer of virus-specific cytotoxic T lymphocytes (VSTs) was postulated to be able to reconstitute specific immunity and to eliminate virus infected cells [40,41]. T cell immunology directed attention to cytotoxic T lymphocytes (CTLs), which play an important role in the immune system defense against viral infections and malignancies [42,43,44,45,46,47]. CTLs are triggered by the presentation of antigen peptides in the context of major histocompatibility complex (MHC) class I molecules, leading to target cell death [44,48,49,50]. The success of VSTs in treating severe viral infections led to the idea of banked third party VSTs, as an approach to rapid treatment of intractable viral infections after allo-HSCT [51,52,53,54,55]. Although CTLs protect us from viral and bacterial infections by destroying infected cells, this process requires T cell MHC allele recognition, which is known as MHC restriction.

However, the recent discovery of induced pluripotent stem cells (iPSCs) [56,57], along with gene editing using CRISPR/Cas9 technology, has cast light upon the road leading toward allogeneic cancer immunotherapy.

In this review we discuss new treatment strategies using iPSC technology for cancer immunotherapy, with suggestions on their prospects.

## 2. Adoptive T Cell Therapy and Tumor Microenvironment

Adoptive T cell therapy, with administration of antigen-specific CTLs expanded ex vivo, has shown success in infections caused by a variety of viruses [58,59,60,61,62] and in malignancies such as melanoma [63,64,65,66,67,68]. However, solid tumors are relatively insusceptible to T cell-mediated destruction in the tumor microenvironment, which is an obstacle when using adoptive T cell therapy against solid tumors [68,69].

Cancer cells utilize both passive and active defense mechanisms to survive [70]. A passive defense mechanism is the reduction of MHC expression or antigen processing ability to limit antigen detection by T cells or the editing of presented antigens by not expressing targeted antigens [71]. An active defense mechanism, by contrast, directly limits the cytotoxic capacity of T cells. For example, they may express Fas ligand that induces apoptosis in CTLs expressing the cognate receptor [72,73,74], or they may secrete soluble factors such as transforming growth factor beta and interleukin(IL)-10 [75,76,77,78] as well as chemokines that attract regulatory T cells which, in turn, inhibit effector T cell function [79,80]. Other elements, such as programmed death receptor-1(PD-1), also inhibit the proliferation and activation of effector T cell function through the recruitment of SHP2, which inactivates T cell receptor-mediated signaling [81,82,83,84].

## 3. T Cell Exhaustion

These passive and active defense mechanisms can lead to CTL exhaustion and deactivation. T cell exhaustion is a state in where T cell dysfunction arises in association with chronic infection and cancer [85,86]. It is defined by poor effector function, sustained expression of inhibitory receptors and an altered transcriptional program [85]. This prevents optimal control of infections and tumors, leading to apoptosis of exhausted cells. However, revitalizing exhausted T cells can reinvigorate immunity [86].

## 4. Use of iPSC Technology to Reinvigorate Immunity

iPSC technology effectively overcomes T cell exhaustion and bolsters immunity. iPSCs were created in 2006 [56,57]; iPSC technology has since been widely utilized in the medical field. 2014 saw the first clinical trial of autologous iPSC-derived cell therapy, in which iPSC-derived retinal pigment epithelial cells were used to palliate wet-type age-related macular degeneration [87,88]. In 2019, allogeneic iPSC-derived corneal epithelial cells were transplanted into a patient with corneal epithelial stem cell deficiency [89].

Recently, iPSC technology has been utilized in cancer immunotherapy [45,46,47].

## 5. Rejuvenation of CTLs

In 2013, a new strategy was created to solve the problem of T cell exhaustion by converting exhausted antigen-specific CTLs into iPSCs (T-iPSCs) [90]. This involved transducing two Sendai virus vectors, along with the Yamanaka four factors [56] and SV40 large T antigen, into a Nef-specific CTL clone derived from the peripheral blood of a human immunodeficiency virus-infected patient. These rejuvenated CTLs showed the same antigen specificity as the original CTLs throughout the whole process of reprogramming, redifferentiation, and expansion. They also proliferated better and had longer telomeres than the original CTLs, and they did not express exhaustion markers such as PD-1 [90]. Almost simultaneously, CTLs specific for melanoma epitope MART-1 were created. These secreted interferon γ, demonstrating that redifferentiation into functional MART-1-specific CTLs had occurred [91]. These findings paved the way for use of iPSC-derived CTLs in viral and malignant diseases.

## 6. Anti-Tumor Effect of iPSC-Derived CTLs Proven In Vivo

iPSC-derived rejuvenated CTLs applied in vivo, using EBV-specific CTLs, were first described in 2015; treatment with rejuvenated CTLs significantly prolonged survival of mice harboring EBV-infected lymphoma cells [92].

In 2020, iPSC-derived EBV antigen-specific CTLs directed against a highly aggressive lymphoma, extranodal natural killer (NK)/T cell lymphoma and nasal type (ENKL), showed promising results that included significantly prolonged survival, robust tumor suppressive effects, and persistence of administered cells as central memory T cells in vivo for at least 6 months, providing a sustainable effect [93]. ENKL cells infected by EBV express a small number of EBV genes, including those encoding latent membrane protein (LMP)1 and/or LMP2, used as an antigen target [58,61,94,95,96,97].

## 7. Rejuvenated CTLs for Solid Cancers

Rejuvenated CTLs have also shown promising results in other malignancies, including cervical cancer associated with human papilloma virus (HPV) infection [98] and renal cell carcinoma [99]. For cervical cancer, T-iPSCs established from HPV16 E6-or E7-CTLs efficiently differentiated into HPV16-specific rejuvenated CTLs; rejuvenated CTLs survived longer than original CTLs, while inducing increased tumor shrinkage and substantially prolonged survival in a mouse model in vivo [98]. For renal cell carcinomas, T-iPSC established from Wilms tumor 1-specific CTLs suppressed tumor growth in a mouse model in vivo [99]. Furthermore, T-iPSC technology has shown success against Ewing sarcoma [100]; rejuvenated CTLs directed against neoantigen encoded by the EWS/FLI1 fusion gene showed an anti-tumor effect in xenograft mouse models.

## 8. Rejuvenated CTLs as an Unlimited Source for “Off-the-Shelf” Therapy

iPSC-derived autologous CTLs are impracticably expensive to generate. Furthermore, their generation takes time—time that patients with rapidly progressing disease do not have. Allogeneic banking of third-party T-iPSCs using antigen specific CTL clones established from peripheral blood of healthy donors could be a solution for both problems [101]. Antigen-specific CTLs could be used to make various antigen-specific rejuvenated CTLs available whenever needed. While many trials using banked third-party peripheral blood-derived virus specific T cells have already shown success [51,52,53,54], use of such cells is limited by potential for expansion ex vivo. Rejuvenated CTLs proliferate strongly and could constitute an unlimited source of antigen-specific T-iPSCs to permit “off-the-shelf” therapy for patients around the world.

In establishing a T-iPSC third-party bank, the burdens of the HLA barrier must be overcome [102]. In order to do so, one must address, from a global perspective, how many T-iPSC lines are necessary to provide rejuvenated CTLs for patients around the world. In a multicenter trial, 32 virus-specific T cell lines were produced, and 18 were applied to 50 study patients. Specifically selected T cell lines matched one or more HLA alleles in 90% of the selected patients [51,52,53,54,55,101]. This suggests an approximate number of cell lines needed to cover a large population, and T-iPSC banking for “off-the-shelf” therapy could be patterned similarly.

## 9. iPSC Technology Exerting Its “Off-the-Shelf” Advantage in CART Therapy

**Figure 1:**
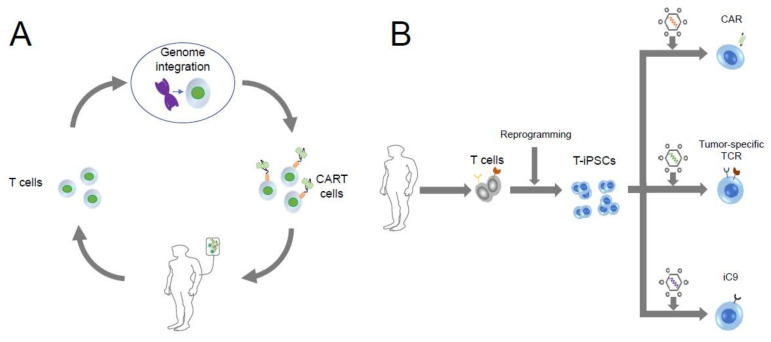
Autologous CART therapy and iPSC-derived engineered T cell therapy. (**A**) T cells are extracted from patients by apheresis and CARs are integrated into T cells, then re-infused to the patient. (**B**) After T cells are reprogrammed into T-iPSCs, T-iPSCs can be transduced with CARs, TCRs, suicide gene iC9, etc.

CARs have also emerged as an innovative strategy for adoptive T cell therapy [103,104,105,106,107] free of HLA restrictions, meaning that cells expressing CARs can be used regardless of patient HLA types [108,109,110,111,112] (Figure 1A). Clinical trials of CART therapy directed against the antigen CD19 have shown nearly 90% complete remission in patients with relapsed or treatment-refractory acute lymphoblastic leukemia [113,114,115,116].

Although patient-derived CART therapy shows promising results, autologous CART cells are individually manufactured, meaning that quantity and quality of the cells administered are sometimes problematic. From blood collection to administration of adequate doses of an autologous CART product, it may take three or more weeks, too long for patients with rapidly progressing disease [70]. Autologous T cells exhausted by chronic exposure to tumor cells often migrate and proliferate poorly, making them unable to eradicate tumors [85,117,118,119,120,121]. “Off-the-shelf” CART therapy may offer promise in meeting these needs. Table 1 lists various strategies in allogeneic CART therapy. Unless using MHC-unrestricted cells when engrafting CARs, such as γδ T cells and NK cells, which are known to be less likely to trigger GVHD, genome editing is indispensable in allogeneic CART therapy. iPSCs are considered suitable for genetic modification, and the CAR transgene can be effectively introduced into iPSCs. Such iPSC-derived CART cells directed against CD19 antigen shrank CD19-expressing lymphoma cell lines in a mouse model [122,123]. The combination of CARs and rejuvenated CTLs (dual antigen rejuvenated CTLs; DRrejTs) was used against treatment refractory EBV-associated lymphoma, where a CAR was engineered to target the CD19 antigen and introduced into iPSC-derived from latent membrane protein 2-specific CTLs to generate DRrejTs. This strategy showed a robust anti-tumor effect and long persistence against human lymphoma in an immunodeficient mouse model. It may soon help to overcome several substantial problems for the treatment of refractory lymphoma patients [124] (Figure 1B).

## 10. Suicide Gene-Based Safeguard System

**Figure 2:**
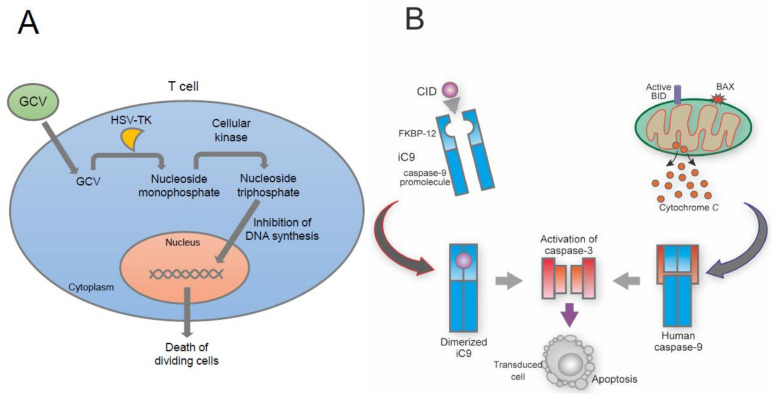
Expression of suicide genes in T lymphocytes. (**A**) HSV-TK phosphorylates ganciclovir (GCV) to nucleoside monophosphate, then a second cellular kinase phosphorylates this into nucleoside triphosphate. This inhibits DNA synthesis, resulting in death of dividing cells. (**B**) In the mitochondrial apoptosis pathway, translocation of activated BAX or BID to the mitochondria creates pores and allows cytochrome C to move to the cytosol. Cytochrome C then activates caspase-9, resulting in the activation of caspase-3, which leads to apoptosis. iC9 was made by the fusion of human caspase-9 to a modified FK506-binding protein (FKBP12), allowing dimerization. By adding CID, iC9 is dimerized and activates caspase-3 directly, also inducing apoptosis.

Gene-engineered T cells react strongly to tumor-associated antigens. This is desirable. A drawback, however, is that if such an antigen is expressed on healthy tissue, these T cells might attack that tissue, causing off-tumor toxicity along with cytokine storm [108,125,126]. These concerns have increased interest in introducing suicide genes into donor cells. The first suicide gene deployed was herpes simplex virus-thymidine kinase (HSV-TK) [127,128,129] (Figure 2A). A step forward has come with the introduction of inducible caspase-9 (iC9) protein, fused to human caspase-9 and to modified FK506-binding protein, which has overcome several concerns associated with HSV-TK [130,131,132,133,134,135,136,137,138] (Figure 2B) and has shown an effect against GVHD in leukemia [134,136,137]. This iC9 safeguard system has also shown effectiveness and safety in regenerative medicine [139,140,141,142,143]. In the treatment of a spinal cord injury, although transplantation of iPSC-derived neural stem/progenitor cells was a promising approach to amelioration of motor function, teratoma formation was a major concern. This was resolved by utilizing the iC9 safeguard system to ablate all transplanted iPSC-derived cells that hindered legitimate motor function [142]. CRISPR/Cas9 genome editing refined the system to introduce a second drug-inducible safeguard: knocking in an iC9 immediately downstream of NANOG allowed killing only iPSCs to prevent teratoma formation, whereas knocking an iC9 into *ACTB* allowed elimination of all cells [24,70]. This iC9 safeguard system has been used in preclinical trials for rejuvenated CTL therapy. These showed that iPSC-derived rejuvenated CTLs exerted a strong antitumor effect, and that introduction of the iC9 safeguard system did not disturb antigen-specific killing activity [92]. The system has also shown promise in CART therapy, where cytokine release syndrome has often been a problem [131,144]. Suicide gene systems based on iC9 are a promising facilitator of clinical use of iPSC-derived cell therapy.

## 11. Gene Edited iPSC-Derived T Cell Therapy

The greatest advantage of allogeneic rejuvenated CTL therapy lies in the ease of delivery from an unlimited source, once T-iPSC banking is established. If the T-iPSC bank is fully stocked, rejuvenated HLA-matched CTLs can be distributed. However, fully stocking the T-iPSC banking may be difficult if serving populations in which HLA haplotypes widely vary. To overcome this problem, HLA engineering has been performed in T-iPSCs from healthy donors, making it possible to create allogeneic CTLs that can be used to treat HLA-matched patients.

To overcome these complications, genome editing is essential. Many methods have been reported [54,145,146,147,148,149,150,151,152,153,154,155]. In immunocompetent patients, rejection by recipient CD8 T cells is the primary complication.

**Figure 3:**
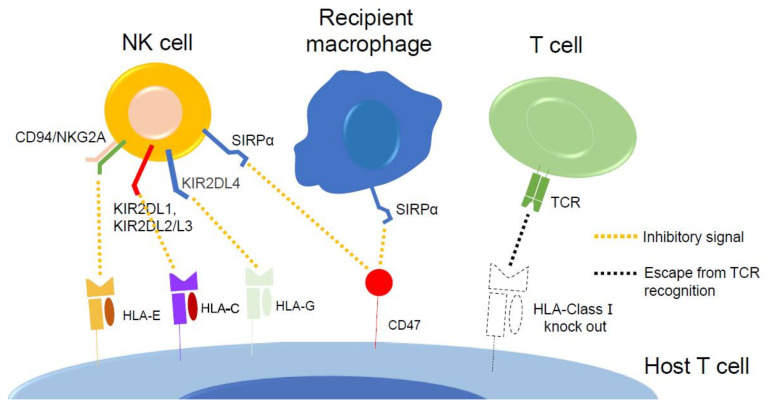
CRISPR/Cas9 gene editing of iPSCs can reduce immune rejection by recipient cells. Deletion of HLA class I by *B2M* knock out prevents T cell rejection. HLA-C and HLA-E protect against NK cell “missing self” response. NK cell attack can also be blocked by the overexpression of CD47.

To address this, *B2M*, encoding β2-microglobulin (*B2M*), a common protein subunit necessary for surface expression of all HLA in class I, has been disrupted. However, disruption of *B2M* causes NK cells to attack HLA class I negative cells because they lack an essential inhibitory ligand, thus triggering the “missing-self” response [54,145,146,147,148,151]. This response was first initially reported in the setting of bone marrow transplantation where MHC-homo mouse cells were rejected by MHC-hetero recipient mice [156,157], a phenomenon eventually traced to NK cells [149,150,158]. To prevent this “missing self” response, many strategies have been assayed. *B2M* was knocked out to erase HLA-class I expression, and *HLA-E* was knocked in. Its product, HLA-E, binds to the CD94/NKG2A receptor on NK cells and protects cells that express HLA-class I from NK-dependent lysis. Thus, the overexpression of HLA-E prevents the “missing self” response [150,151]. In another approach, iPSCs overexpressed CD47, one of the “don’t eat me” signals, after the depletion of HLA molecules [146], which impaired NK cell response in *B2M* knock out cells. Yet another approach deleted iPSC HLA class I and II while knocking in *HLA-G*, *CD47*, and *PD-L1* [152]. This strategy was supported by reports that HLA-G1 reduces the NK cell-mediated immune response [153,154,155] and by the evident fact that PD-L1 suppresses T cell activity. A different group developed HLA-homo-iPSCs in which HLA-A, HLA-B, and HLA-class II were deleted but HLA-C was retained; non-canonical HLA molecules were expected to prevent immune rejection by NK cells [148,150]. Recently, PVR knock out (*PVR* encodes a ligand on the NK cell-activating receptor DNAM-1), HLA-E transduced, HLA-I- and HLA-II null iPSC successfully yielded CTLs that escaped alloreactive host recognition [159] (Figure 3).

Although these techniques are very promising, they risk off-target genome editing, which might cause oncogenic events. Strategies such as single cell genome editing and introduction of a safeguard system can evade this risk. Single cell genome editing of iPSCs is considered technically feasible, and the iC9 safeguard system, as explained earlier, is also promising.

## 12. Conclusions

Innovative progress continues in allogeneic cell cancer immunotherapy, with advances toward clinical use. Banking of third-party T-iPSCs or generation of hypoimmunogenic iPSC by HLA editing, with the banked cells as an unlimited source of therapeutic T cells, would be a feasible approach for “off-the-shelf” therapy available for urgent use at a reasonable cost [51,52,53,54,101]. Introduction of T-iPSC technology into T cell therapy could thus confer better clinical prognosis. Incorporation of the iC9 system constitutes an efficient safeguard for iPSC-derived cell therapy. These approaches are paving the way into a new and promising era for allogeneic immunotherapy in cancer treatment.

## Figures and Tables

**Table 1:** Various strategies in allogeneic CART therapy.

Company	Products	CAR	Allogeneic Cell Source	Strategy to Evade GVHD or Immune Rejection	Target Gene	Clinical Trial Identifier
Allogene Therapeutics	ALLO-501 (in USA)	CD19	T cell	TALEN	TRAC and CD52	NCT03939026
	ALLO-715	BCMA				NCT04093596
	ALLO-316	CD70				NCT04696731
Adicet Bio, Inc.	ADI-001	CD20	γδT cell			NCT04735471
Caribou Biosciences, Inc.	CB010A	CD19	T cell	CRISPR/Cas9	TRAC, PD1 (KO)	NCT04637763
Cellectis	UCART-123	CD123	T cell	TALEN	TRAC (KO)	NCT03190278 (AML) NCT03203369 (BPDCN) NCT04150497 NCT04142619
		
	UCART-22	CD22			TRAC and CD52 (KO)	
	UCART-CS1	CS1			TRAC and CS1 (KO)	
Celyad	CYAD-211	BCMA	T cell	shRNA	CD3ζ	NCT03692429
	CYAD-101	NKG2DL		Overexpression of TIM	-	NCT03692429
CRISPR Therapeutics AG	CTX110	CD19	T cell	CRISPR/Cas9	TRAC and B2M (KO)	NCT04035434
	CTX120	BCMA				NCT04244656
	CTX130	CD70				NCT04502446
Fate Therapeutics	FT-819	CD19	iPSC-derived T cell	CRISPR/Cas9	TRAC	NCT04629729
Great Ormond Street Hospital for Children	PBLTT52CAR19	CD19	T cell	CRISPR/Cas9	CD52 and TRAC (KO)	NCT04557436
Kurr therapeutics	KUR-502	CD19	NKT			NCT03774654
Precision Biosciences	PBCAR0191	CD19	T cell	meganuclease mRNA shRNA	B2M (shRNA), HLA-E	NCT03666000
	PBCAR19B	CD19				NCT04649112
	PBCAR20A	CD20				NCT04030195
	PBCAR269A	BCMA				NCT04171843
Tessa Therapeutics Pte Ltd.	CD30.CAR-EBVST cells	CD30	EBVST			NCT04288726

## Data Availability

Not applicable.
